# Catch of the Day: New Serum Amyloid A (SAA) Antibody Is a Valuable Tool to Study Fish Health in Salmonids

**DOI:** 10.3390/cells12162097

**Published:** 2023-08-19

**Authors:** Ralfs Buks, Abdo Alnabulsi, Rodanthi Zindrili, Ayham Alnabulsi, Alex Wang, Tiehui Wang, Samuel A. M. Martin

**Affiliations:** 1 Scottish Fish Immunology Research Centre, School of Biological Sciences, University of Aberdeen, Aberdeen AB24 2TZ, UK; 2 Vertebrate Antibodies Ltd., Aberdeen AB24 2TZ, UK

**Keywords:** serum amyloid A (SAA), SAA antibody, SAA recombinant protein, RTS-11 cell line, salmonids, research tools, fish health

## Abstract

Serum amyloid A (SAA) proteins belong to a family of acute-phase reactants, playing an integral role in defending the organism from pathological damage. Despite a wealth of data on the regulation of SAA transcripts in teleosts, there is only limited information on these proteins’ abundance in fish. The aim of this study is to characterise SAA protein levels in salmonids using a newly developed antibody specific to salmonid SAA. The salmonid SAA antibody detected SAA and accurately discriminated between stimulated and control specimens from rainbow trout macrophage cell line (RTS-11) in vitro, as well as rainbow trout challenged with *Aeromonas salmonicida*- or flagellin-stimulated Atlantic salmon in vivo. The presence of SAA protein was analysed in RTS-11 cell line supernatants, liver, and spleen samples using ELISA, immunoblotting, and immunohistochemistry. This study is the first to characterise SAA protein levels in salmonids in vivo and in vitro. The newly developed salmonid SAA antibody was able to discriminate between stimulated and unstimulated specimens, showing that it can be used to study the acute-phase response in salmonids with the potential to be further developed into assays to monitor and evaluate health in wild and farmed fish.

## 1. Introduction

Acute-phase proteins (APPs) form a highly conserved part of innate immunity, defending the organism from pathological damage and aiding in restoring homeostasis. Large increases in APPs can be triggered by pathogen invasion, stimulation with strong danger signals, or stress. Serum amyloid A (SAA) proteins are part of a family of closely related and highly conserved serum molecules. SAAs are small (~12 kDa) APPs, which are comprehensively studied in humans and mice [1] and reported in other vertebrates including birds [2] and fish [3,4,5,6].

SAA proteins are divided into two distinct groups: acute-phase SAAs and constitutively expressed SAAs, with the latter group reported exclusively in humans and mice. Upon stimulation, acute-phase SAA genes are induced via a few-fold increase to over three orders of magnitude, suggesting the importance of the SAA proteins in restoring homeostasis. Although a complete list of the functions of APPs is yet to be established, SAAs have multiple roles, including recruitment of immune cells to the inflammatory sites, opsonisation, cholesterol transport, and the induction of enzymes involved in degradation of the extracellular matrix [1,7].

SAAs are predominantly synthesised by hepatocytes in liver, while extrahepatic synthesis has been reported in other tissues, including spleen [8]. At the molecular level, SAA transcription responds to various inducers, including pro-inflammatory cytokines such as interleukin-1 beta (IL-1β), interleukin-6 (IL-6), and tumor necrosis factor alpha (TNFα), as well as lipopolysaccharides (LPS) [4]. Elevated SAA levels typically resolve completely once the initiating stimulus is withdrawn. However, the mechanisms driving this process remain unclear.

SAAs are known APPs in rainbow trout (*Oncorhynchus mykiss*), Atlantic salmon (*Salmo salar*), and other teleosts [9,10,11,12] making these proteins attractive markers by which to monitor fish health status. Atlantic salmon *SAA-5* transcripts were reported as the dominant APP response following *Aeromonas salmonicida* challenge [13]. The majority of studies in fish describe APPs at the gene expression level, primarily due to a lack of commercially available research tools with which to detect SAA proteins. A limited number of studies have characterised the expression of SAA proteins—i.e., by using immunohistochemistry (IHC)—in challenged rainbow trout liver and head kidney samples [10,14]. SAA protein detection using methods other than IHC has proven to be difficult, likely due to SAA association with high density lipoproteins (HDLs) [15]. Despite multiple attempts, the presence of SAA in fish blood has been suggested [14] but not conclusively detected, and its presence and role in circulation still remain unclear [16].

The aim of this study is first to develop an anti-salmonid SAA antibody that could be used as a research tool to study fish health, and second, to characterise the expression of SAA protein in salmonids. After characterisation, the antibody was used to detect SAA protein in flagellin-stimulated Atlantic salmon samples in vivo, RTS-11 cell line in vitro, and *Aeromonas salmonicida*-challenged rainbow trout in vivo. SAA was detected in liver and spleen samples using ELISA, immunoblots, and IHC. The newly developed salmonid SAA antibody was able to discriminate between stimulated and unstimulated specimens, suggesting that it could be used in APP research and to potentially monitor and study the health of farmed fish.

## 2. Materials and Methods

All procedures were carried out under the UK Animals (Scientific Procedures) Act 1986 and the Home Office code of practice guidance under the Home Office project license PFF8CC5BE.

### 2.1. Fish Challenges

Atlantic salmon parr (~38 g) from the recirculating aquaculture system (RAS) hatchery of the Scottish Sea Farms Ltd. (Barcaldine Hatchery, Barcaldine, UK) were introduced to the freshwater facilities of the University of Aberdeen at the School of Biological Sciences. Fish were maintained in a stable water temperature of 12 °C and fed commercial diet at 1.5% average body weight per day (Biomar, Grangemouth, UK). Atlantic salmon (70 ± 14 g) were stimulated with *Yersinia ruckeri* flagellin, a potent immunostimulant [17]. An amount of 50 ng flagellin per gram of body weight [18] or PBS (control) was injected intraperitoneally. Stimulation was allowed to proceed for 6, 24, or 48 h (h) (*n* = 8 fish per timepoint; *n* = 4 stimulated; and *n* = 4 PBS control) prior to sacrifice via overdose with MS-222 (Sigma-Aldrich, St. Louis, MO, USA) followed by destruction of the brain before immediate sampling. Liver and spleen samples were collected from four fish in each group and processed for IHC as described below.

Rainbow trout (~75 g) were purchased commercially, maintained in 1 m diameter fiberglass tanks with recirculating freshwater at 14 ± 1 °C and fed a commercial diet at 1.5% average body weight per day (Skretting, Stavanger, Norway) as previously described [19]. Fish were injected interperitoneally with 100 μL of live *Aeromonas salmonicida* (2 × 10^6^ CFU/mL) or PBS. Rainbow trout (271 ± 22 g) were culled 48 h post-challenge via overdose of 2-phenoxyethanol (Sigma-Aldrich, St. Louis, MO, USA), followed by destruction of the brain prior to immediate sampling. Liver samples were collected from four fish in each group and processed for IHC as described below.

### 2.2. Fish Tissue Preparation

Liver and spleen samples (~5 × 5 × 5 mm) were fixed at room temperature (RT) for 24 h in 10% neutral buffered formalin (CellPath, Newtown, UK). Samples were washed 5 times with PBS and stored in 70% ethanol at 4 °C until histological examination. The preparation of Atlantic salmon liver and spleen samples and rainbow trout liver samples for image acquisition was carried out by the University of Aberdeen Microscopy and Histology Core Facility. Samples were dehydrated, embedded in wax, and cut in 5 μm cross sections ready for IHC.

### 2.3. Production of Recombinant SAAs

The nucleotide sequences encoding mature peptides of SAAs of Atlantic salmon (Acc. No. B9EPA2) and rainbow trout (Acc. No. NP_001117908) were code-optimised for the ex-pression in *E. coli* and synthesised by Eurofins Genomics. The synthesised DNA strands were cloned to a pET/Duet vector (Novagen, Paris, France) via gene assemble and the sequence was confirmed. The DNA constructs encode the complete mature SAAs with a N-terminal his-tag (MAHHHHHHHHGSGS) for purification. The production of the recombinant proteins in *E. coli* BL21 Star (DE3), the purification under denaturing conditions, the refolding, and the quantification of the recombinant proteins were as described previously [20].

### 2.4. SAA Monoclonal Antibody Production

**Figure 1:**
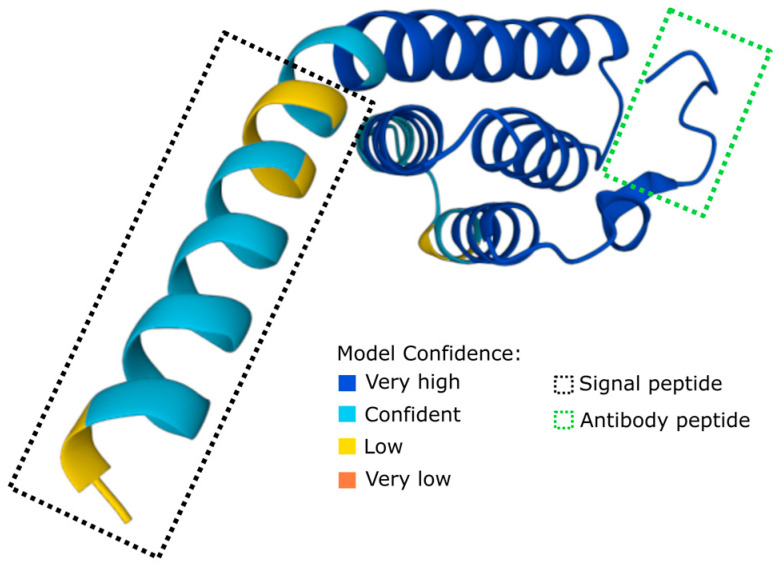
AlphaFold-predicted 3D structure of the SAA-5 protein (UniProt B9EPA2). AlphaFold produces a per-residue confidence score (pLDDT) between 0 and 100. Model Confidence is categorised as very high (pLDDT > 90), confident (90 > pLDDT > 70), low (70 > pLDDT > 50), or very low (pLDDT < 50). Black dotted rectangle denotes predicted signal peptide of the SAA-5 (MKLLLAGLVLTLVVGAQA). Green dotted rectangle highlights the peptide sequence (RYRPNGLPRNY) used as the immunogen for the SAA antibody development.

The optimal immunogen properties were chosen as previously described [21]. The SAA peptide sequence (RYRPNGLPRNY) was selected from Uniprot B9EPA2 as an immunogen due to its unique properties and analysis by the Immune epitope database (IEDB) and AlphaFold [22,23]. The peptide at the C-terminal of the Atlantic salmon SAA-5 protein (Figure 1) was predicted to be linear, accessible, hydrophilic, and located on the surface. To confirm its uniqueness and avoid potential cross-reactivity, the peptide was subjected to a basic local alignment search tool (BLAST) analysis [24,25]. The selected peptide was then synthesised by Almac Sciences Ltd. and conjugated to ovalbumin (OVA) for immunization. The procedure for generating the monoclonal antibodies (mAbs) was performed as described previously [26]. The resulting hybridomas were screened against the peptide conjugated with bovine serum albumin (BSA) by enzyme-linked immunosorbent assay (ELISA). Hybridoma clones showing a strong positive signal to the immunogen peptide were then subcloned to ensure monoclonality. The positive hybridoma clones were grown in Hybridoma-SFM (Thermo Fisher Scientific, Waltham, MA, USA) to produce working stocks of antibodies. The selected clone A10 of the anti-salmonid SAA mAb was purified via affinity chromatography using protein G Plus/protein A-Agarose (Milipore, Burlington, MA, USA) and conjugated with NHS-LC-Biotin (Thermo Fisher Scientific, Waltham, MA, USA).

### 2.5. SDS-PAGE Immunoblots

Recombinant Atlantic salmon SAA protein (10 or 40 ng), recombinant rainbow trout SAA protein (40 ng), and supernatants (20 μL) from IL-1β-stimulated or control RTS-11 cells were used and were mixed with NuPAGE LDS loading buffer (Invitrogen, Waltham, MA, USA) containing 5% β-mercaptoethanol (β-ME, Sigma-Aldrich, St. Louis, MO, USA), incubated at 70 °C for 10 min, size separated in a 4–12% Bis-tris SDS-PAGE gel (Invitrogen, Waltham, MA, USA), and transferred to PVDF membrane (Amersham™, Merck, Rahway, NJ, USA). SAA protein was detected using the newly developed anti-SAA antibody and an anti-mouse-HRP-conjugated antibody (Sigma-Aldrich, St. Louis, MO, USA). The signal was revealed using ECL substrate (Biorad, Hercules, CA, USA) with GenoPlex system (VWR, Radnor, PA, USA) and quantified using Fiji v2.9.0 (NIH, Bethesda, MD, USA).

### 2.6. Atlantic Salmon rSAA Detection by Indirect ELISA

Recombinant Atlantic salmon SAA protein was diluted in L-15 media (Gibco™, Thermo Fisher Scientific, Waltham, MA, USA). An amount of 100 μL of L-15 media (background control), along with 0.2, 0.5, 1, 5 and 10 ng rSAA diluted in L-15 media, were added to 96-well ELISA plates (Greiner Bio-One, Kremsmünster, Austria) and incubated at 4 °C overnight. Wells were blocked with 300 μL of 1% BSA (Sigma-Aldrich, St. Louis, MO, USA) in PBS for 1 h at RT. The anti-SAA biotin-linked antibody was diluted in 1% BSA + 0.05% Tween (Sigma-Aldrich, St. Louis, MO, USA) and 100 μL (75 ng/well) was added to each well. After 1 h incubation at RT, wells were washed three times with 300 μL PBS + 0.05% Tween (PBST). A total of 100 μL of poly-HRP (1:20,000 in PBST) was added, and the plate was incubated at RT for 30 min. The plate was washed five times with PBST, followed by the addition of 50 μL TMB substrate (Abcam, Cambridge, UK) for 30 min at RT. The reaction was stopped with 50 μL 2 M H_2_SO_4_ (Sigma-Aldrich, St. Louis, MO, USA), after which the plate was read at 450 nm using a SpectraMax^®^ ABS Plus spectrophotometer (Molecular Devices, San Jose, CA, USA).

### 2.7. RTS-11 Stimulation with Recombinant Rainbow Trout IL-1β

A mononuclear/macrophage-like rainbow trout cell line, RTS-11 [27], was cultured at 20 °C in Leibovitz L-15 media supplemented with 30% fetal calf serum (FCS) and 1% Penicillin/Streptomycin (P/S) (ThermoFisher Scientific, Waltham, MA, USA). Cells were washed once with PBS and 1 million cells/mL were seeded into 24-well plates in L-15 medium with P/S and without FCS. Prior to stimulation, cells were incubated for 24 h at 20 °C. RTS-11 cells were stimulated with 25 ng/mL IL-1β, a well described SAA gene inducer [28]. Cell suspension was collected at 4, 6, 8, 10, 12, 24, 36, 48, and 72 h post-stimulation, centrifuged once, and supernatants were harvested and stored at −20 °C.

### 2.8. Proteomic Analysis of Secreted Proteins from Cell Culture

Supernatant (20 μL) from RTS-11 cells stimulated with 25 ng/mL IL-1β for 36 h was size separated in a 4–12% Bis-tris SDS-PAGE gel as described above. Supernatant sample equivalent to ~11 kDa to ~14 kDa was cut out from the gel and stored in double-distilled water at 4 °C. Proteins were digested in gel using a JANUS automated liquid-handling workstation (PerkinElmer, Beaconsfield, UK) with a customised method adapted from that of Shevchenko and colleagues [29]. Tryptic peptides were dried by vacuum centrifugation and dissolved in 0.1% TFA for liquid chromatographic–tandem mass spectrometric (LC-MS/MS) analysis using a Q Exactive Plus/UltiMate 3000 RSLCnano system (Thermo Fisher Scientific, Hemel Hempstead, UK) equipped with an EASY-Spray source and configured for pre-concentration onto a 25-cm long PepMap RSLC C18 analytical column (Thermo Fisher Scientific). Tandem mass spectra were acquired using a data-dependent “Top 10” method as described before [30]. Peptides were identified by comparison with the *O. mykiss* proteome (UniProt proteome UP000193380, downloaded 17 January 2019) using Proteome Discoverer (version 2.2, Thermo Scientific) and a workflow incorporating the Mascot search engine (version 2.6, Matrix Science, London, UK) and decoy database validation with a strict target false discovery rate (FDR) of 0.01.

### 2.9. Quantification of Secreted SAA Protein in Stimulated RTS-11 Cells by Indirect ELISA

Supernatants (100 μL) from RTS-11 IL-1β stimulation and control 4, 6, 8, 10, 12, and 24 h post-stimulation were used. An amount of 100 μL of L-15 media (Gibco™, Thermo Fisher Scientific, Waltham, MA, USA) (background), along with 0.2, 0.5, 1, 5 and 10 ng recombinant SAA (rSAA) diluted in L-15 media, served as positive control and was used for the calibration curve. Samples were added to 96-well ELISA plates (Greiner Bio-One, Kremsmünster, Austria) and incubated at 4 °C overnight. Wells were blocked with 300 μL of 1% BSA (Sigma-Aldrich, St. Louis, MO, USA) in PBS for 1 h at RT. The anti-SAA biotin-linked antibody was diluted in 1% BSA + 0.05% Tween (Sigma-Aldrich, St. Louis, MO, USA) and 100 μL (75 ng/well) was added to each well. After 1 h incubation at RT, wells were washed three times with 300 μL PBS + 0.05% Tween (PBST). A total of 100 μL of poly-HRP (1:20,000 in PBST) was added, and the plate was incubated at RT for 30 min. The plate was washed five times with PBST followed by the addition of 50 μL TMB substrate (Abcam, Cambridge, UK) for 30 min at RT. The reaction was stopped with 50 μL 2 M H_2_SO_4_ (Sigma-Aldrich, St. Louis, MO, USA), after which the plate was read at 450 nm using a SpectraMax^®^ ABS Plus spectrophotometer (Molecular Devices, San Jose, CA, USA).

### 2.10. IHC and Histological Examination

Before performing the IHC, the sections were dewaxed in xylene, rehydrated in decreasing ethanol concentrations, then washed with tap water. The IHC was performed using a Dako autostainer E 172,566 (Model: LV-1, Dako universal staining system, Dako/Agilent Technologies LDA UK Limited, Cheshire, UK), as previously described [21], with the following modifications: First, the antigen retrieval was performed using Dako PT Link (Pre-Treatment Module for Tissue Specimens, Dako/Agilent Technologies LDA UK Limited, Cheshire, UK). The pre-treatment was set as recommended by Dako; the module was heated to 65 °C, slides were placed into the module, and heat was increased to 97 °C and held at this temperature for 20 min; then, it was cooled to 65 °C before the slides were taken out from the module. Second, liver and spleen slides were incubated with anti-salmonid SAA mAb supernatant (neat) for 60 min at RT. Slides were washed twice with Dako washing buffer. The endogenous peroxidase activity was blocked by incubation with hydrogen peroxidase-blocking solution for 7 min (Dako). The slides were then washed twice using Dako-washing buffer and incubated with peroxidase polymer-labelled goat anti-mouse/rabbit secondary antibody (Dako EnVision™ FLEX Detection system, Cheadle, UK) for 30 min at RT. The stained sections were examined with a Zeiss Axioscope 5 upright microscope (Zeiss, Jena, Germany).

The staining area of SAA was measured using Fiji v2.9.0 (NIH, Bethesda, MD, USA), as performed before [31], with some modifications. Each slide was analysed at 200× magnification for each type of organ, fish, and timepoint (*n* = 4). Briefly, high-resolution Zeiss Axioscope 5 images were subject to colour deconvolution using the H DAB vector, designed to analyse IHC signal obtained from 3, 3′-diaminobenzidine (DAB) and Hematoxylin (H). A brown colour panel was used for SAA analysis. The background for the DAB staining in the IHC image was removed by setting the minimum and maximum threshold to 0 and 175, respectively. Signal area covered by the positive signal was measured as the percentage of cover per image.

### 2.11. Statistical Analysis

The Mann–Whitney test was used for statistical analysis using Prism 5 (GraphPad, San Diego, CA, USA), where ns—not significant, * *p* < 0.05; **** *p* < 0.0001. If not stated otherwise, data are presented as the mean value with standard deviation (SD).

## 3. Results

### 3.1. SAA Analysis in Atlantic Salmon

There are three Atlantic salmon serum amyloid A genes and one SAA gene entry (National Centre for Biotechnology Information (NCBI) library). Serum amyloid A-5 protein (SAA-5) (Gene ID: 100286626, NC_059467.1 (25196317..25197512)) and SAA-5 protein-like (Gene ID: 123730763, NC_059467.1 (25215784..25217197)) are both located on the chromosome 26 [32]. Both share 100% amino acid (aa) sequence identity to the SAA-5 protein (Acc. No. B9EPA2). The protein-like SAA-5 (Gene ID: 123733077, NW_025548462.1 (13210...13892, complement)) has no chromosomal annotation, and it shares 41% amino acid sequence identity with the SAA-5. Lastly, serum amyloid A-like 1 protein (ID: 106584762, NC_059464.1 (45974902..45994216, complement)) is located on chromosome 23 [33].

Previous reports show that the Atlantic salmon SAA-5 is an inducible APP [11], and for this reason, the SAA-5 protein (accession B9EPA2) was chosen as the target for Atlantic salmon SAA antibody development. The 121-aa-long SAA-5 protein has a predicted 19-aa signal peptide on the N-terminus (MKLLLAGLVLTLVVGAQAQ) (Figure 1 and Figure S1). The accessible 11-amino-acid sequence (RYRPNGLPRNY) located on the C-terminus was used as the immunogen for the SAA antibody development (Figure 1A and Figure S1). Recombinant Atlantic salmon SAA-5 protein (aa 20–121) was expressed and used as a positive control to test the reactivity of the produced SAA-5 antibodies.

### 3.2. Anti-Salmonid SAA Monoclonal Antibody Clone A10 Characterisation

The SAA antibody-producing hybridomas were screened against the immunogen peptide sequence (Uniprot B9EPA2) of 11 amino acids (RYRPNGLPRNY) conjugated with bovine serum albumin (BSA) by enzyme-linked immunosorbent assay (ELISA). The A10 clone showed a strong positive signal (OD = 1.530, SD = 0.14) and was therefore selected for this study. The anti-salmonid SAA mAb (Clone A10) was isotyped using an Isostrip kit (Roche Diagnostics, Basel, Switzerland) and confirmed to be an IgG2b isotype with κ light chains.

### 3.3. Anti-SAA A10 Antibody Detects Recombinant Atlantic Salmon SAA Protein by Western Blotting and ELISA

**Figure 2:**
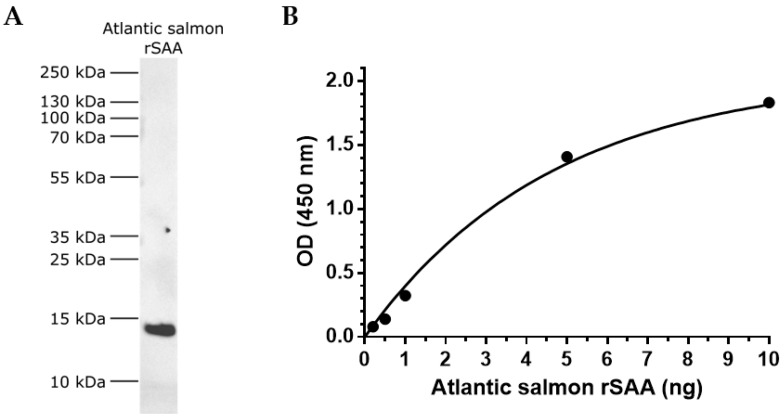
Anti-SAA A10 antibody detection of recombinant Atlantic salmon SAA-5 protein via Western blotting and ELISA. (**A**) A total of 40 ng of Atlantic salmon rSAA-5 was size separated in a 4–12% (*w*/*v*) Bis-Tris SDS-PAGE under reducing conditions and immunostained with anti-SAA A10 monoclonal antibody. (**B**) The 96-well plate was coated with Atlantic salmon rSAA-5 (0.2–10 ng) and the optical density from the anti-SAA-5-biotin-linked antibody was measured via an indirect ELISA. Data are fitted to a one-phase exponential association and each datapoint is presented as mean (*n* = 3) with SD.

First, the SAA A10 antibody was tested using an immunoblot. The SAA antibody recognised a strong single band of the recombinant Atlantic salmon SAA protein at the expected ~13 kDa size (Figure 2A). Second, the SAA antibody was tested to detect a range of the Atlantic salmon rSAA protein levels by ELISA. The SAA antibody was able to discriminate the signal from the background at as low as 0.2 ng rSAA quantity (OD_0.2ng_ = 0.080, SD = 0.002; OD_background_ = 0.045, SD = 0.001) (Figure 2B). Altogether, the data show that the SAA antibody detects Atlantic salmon rSAA protein in reducing conditions as well as at the conformational form.

### 3.4. Analysis of SAA Protein Expression in Atlantic Salmon Liver and Spleen Using IHC following Flagellin Stimulation

**Figure 3:**
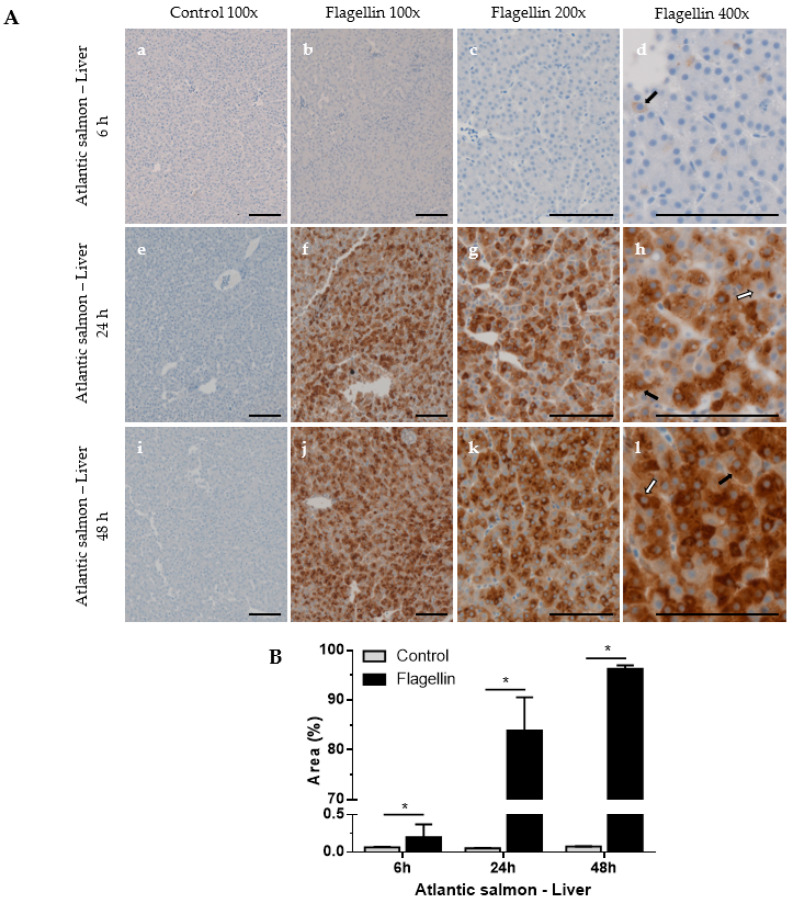
Localisation and analysis of SAA in Atlantic salmon liver using IHC. Fish were stimulated with a flagellin or PBS (control). Liver samples were collected 6, 24, or 48 h post-stimulation, and IHC was performed using anti-SAA A10 antibody. (**A**) Representative images of control (100× magnification, (**a**,**e**,**i**)) and flagellin-stimulated fish (100× (**b**,**f**,**j**), 200× (**c**,**g**,**k**) and 400× magnification (**d**,**h**,**l**)) are shown. Scale bars represent 100 μm. Black arrows represent granulated cytoplasmic SAA, while white arrows denote homogenous SAA staining. (**B**) Area (%) covered by SAA staining measured at 200× magnification. Mann–Whitney test, mean with SD, *n* = 4, * *p* < 0.05.

**Figure 4:**
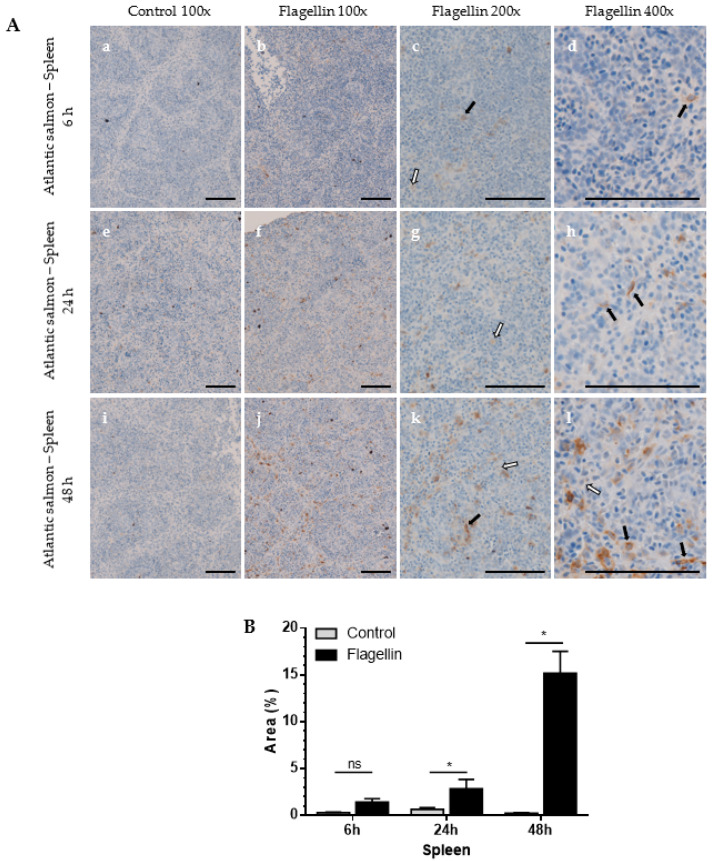
Localisation and analysis of SAA in Atlantic salmon spleen using IHC. Fish were stimulated with a flagellin or PBS (control). Liver samples were collected 6, 24, or 48 h post-stimulation and IHC was performed using the anti-SAA A10 antibody. (**A**) Representative images of control (100× magnification, (**a**,**e**,**i**)) and flagellin-stimulated fish (100× (**b**,**f**,**j**), 200× (**c**,**g**,**k**) and 400× magnification (**d**,**h**,**l**)) are shown. Scale bars represent 100 μm. Black arrows represent granulated cytoplasmic SAA, while white arrows denote homogenous SAA staining. (**B**) Area (%) covered by SAA staining measured at 200× magnification. Mann–Whitney test, mean with SD, *n* = 4. ns—not significant, * *p* < 0.05.

The anti-SAA A10 antibody was used to analyse SAA in Atlantic salmon liver (Figure 3) and spleen (Figure 4) after flagellin stimulation using IHC.

Liver. Control fish showed no signal in liver samples (Figure 3A(a,e,i)). Low SAA levels were observed in liver as early as 6 h post-stimulation, comparable to the control fish (Figure 3A(a–d)). The SAA levels in liver highly increased in the following timepoint where the positive SAA signal covered 84% (SD = 6.7) of the section area at the 24 h timepoint, increasing to 96% (SD = 0.7) 48 h post-stimulation (Figure 3A,B). The continuous increase and a very strong SAA signal suggest the specificity of the anti-SAA A10 antibody and potency of the flagellin used.

Spleen. Similar to liver cells, SAA levels in stimulated spleen showed very low signal 6 h post-stimulation (Figure 4A(a–d)). The SAA levels in spleen showed a modest increase 24 h post-stimulation (Figure 4A(d,h)), followed by further increase 48 h post-stimulation (Figure 4A,B). Control fish showed a very weak signal in spleen samples 6 h post-stimulation (Figure 4A(a,e,i)), suggesting low levels of continuously expressed SAA in spleen in a few cells.

There were both granulated and homogenous stained SAA cells in liver and spleen samples, with the granulated type increasing in later timepoints. The observation suggests that SAA is expressed in specific cytoplasmic compartments, or that a high abundance of SAA forms polymeric structures. Altogether, the data show that the SAA antibody can differentiate between rest and salmonids activated via bacterial infection or flagellin stimulation.

### 3.5. SAA A10 Antibody’s Reactivity to Other Salmonid SAA Proteins

To test the potential Atlantic salmon SAA A10 antibody’s reactivity to other salmonid SAA proteins, the Atlantic salmon SAA-5 protein was subject to BLASTp protein alignment in salmonids (taxid: 8015) using the non-redundant protein sequence database [24]. Surprisingly, there were multiple amino acid differences among salmonids at the SAA C-terminus (Table 1, Figure S1), the region used for antibody development.

**Figure 5:**
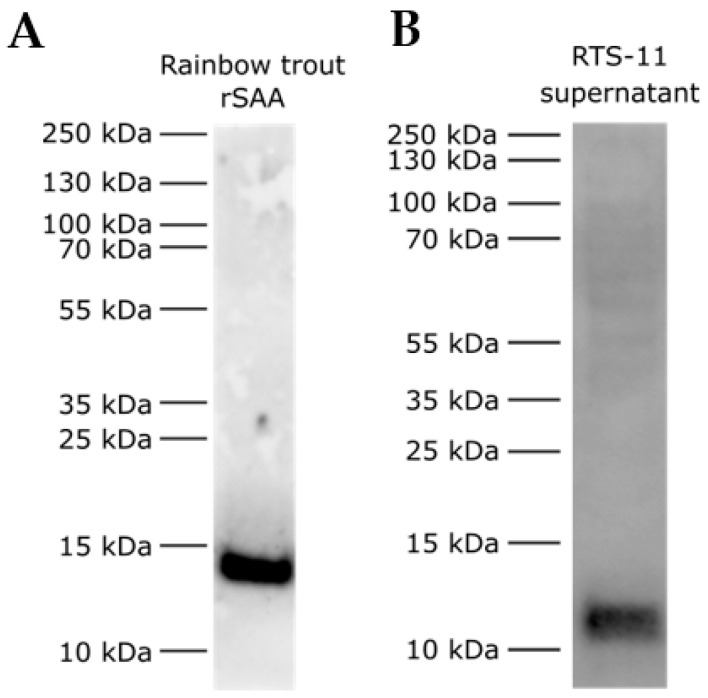
Recombinant rainbow trout SAA and endogenous SAA immunoblot detection by the new salmonid SAA antibody. (**A**) Recombinant rainbow trout SAA protein (40 ng); (**B**) 20 μL of IL-1β-stimulated RTS-11 cell line supernatant were size separated in a 4–12% (*w*/*v*) Bis-Tris SDS-PAGE under reducing conditions and immunostained with the anti-salmonid SAA antibody.

Considering the sequence differences, we expressed a recombinant rainbow trout SAA protein (NP_001117908.1, aa 19 – 121) and tested the SAA A10 antibody’s reactivity to the recombinant protein. The antibody showed a strong signal to the recombinant rainbow trout SAA protein in Western blot (Figure 5A). Furthermore, the SAA A10 antibody detected a strong signal protein at ~12 kDa in the rainbow trout RTS-11 cell line supernatant sample stimulated with IL-1β, suggesting the antibody’s recognition to endogenous secreted SAA (Figure 5B). RTS-11 supernatant proteins and peptides corresponding to ~11 kDa to ~14 kDa were cut out from the SDS-PAGE gel and were subject to proteomic analysis. Two isoforms of the SAA were detected in the sample, further suggesting the specificity of the antibody to endogenous SAA (Table S1). A total of 40 proteins were detected, out of which 5 (12.5%) were uncharacterised rainbow trout proteins (Table S1). SAA was quantified as the seventh most abundant protein (Table S1).

These results demonstrate that the developed SAA A10 antibody recognises SAA-5 from *Salmo salar* (Figure 2) and SAA in *Oncorhynchus mykiss* (Figure 5). Based on the sequence identity of the two recombinant SAA proteins used in this study, the SAA A10 antibody is expected to recognise SAA in *Salmo salar*, *Salmo trutta*, *Oncorhynchus mykiss*, *Oncorhynchus keta*, *Oncorhynchus kisutch*, *Oncorhynchus gorbuscha*, *Oncorhynchus tshawytscha*, and *Oncorhynchus nerka* (Table 1).

### 3.6. Temporal Production of SAA Protein following IL-1β Stimulation in RTS-11 Cells

**Figure 6:**
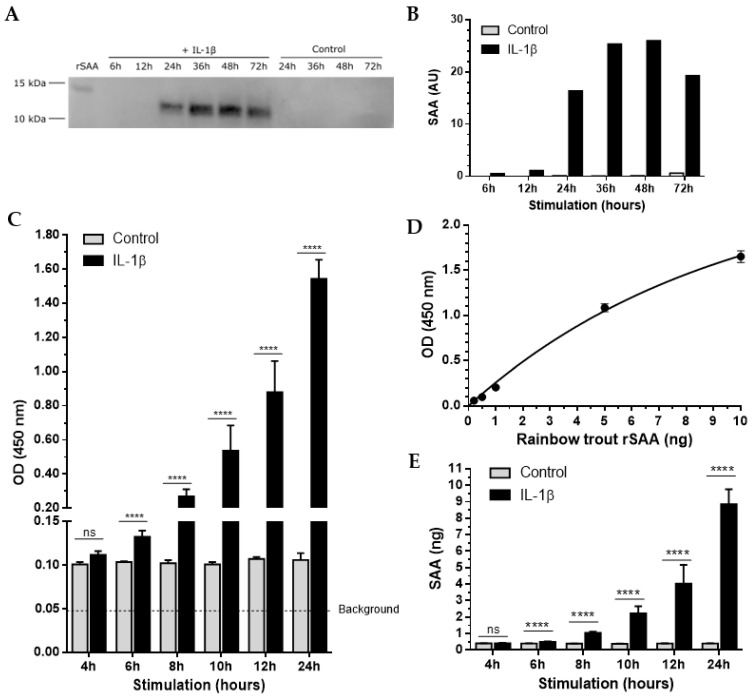
SAA protein is synthesised early following IL-1β stimulation in the RTS-11 cell line. (**A**) SDS-PAGE immunoblots of SAA in RTS-11 cell supernatants 6, 12, 24, 48, and 72 h post-stimulation with IL-1β or PBS (control). Rainbow trout rSAA (10 ng) was used as a positive control. (**B**) Densitometry plot comparing SAA protein abundance from the SDS-PAGE immunoblot (**A**): grey (control), black (IL-1β) stimulation. (**C**) Optical density (OD) of SAA protein levels in supernatants from RTS-11 control (grey) and IL-1β (black)-stimulated cells for 4, 6, 8, 10, 12, and 24 h measured using indirect ELISA. Background signal (OD = 0.048) from the RTS-11 culture media without cells is noted by the black dashed line. The Y-axis is divided into two segments visualising the SAA protein level differences 4 and 6 h post-stimulation. (**D**) Representative standard curve of OD values from 0.2, 0.5, 1, 5, and 10 ng rainbow trout rSAA protein. *n* = 3, mean with SD. (**E**) Estimated SAA extracellular protein levels in 100 μL RTS-11-stimulated media based on mean OD values (**C**) and the standard curve (**D**). RTS-11 cells were stimulated in three independent experiments. Each sample was measured in duplicates by ELISA. Data are presented as the means with SDs, Mann–Whitney test. ns—not significant, **** *p* < 0.0001.

IL-1β is known to induce *SAA* transcription in rainbow trout cells [34,35]. However, SAA protein levels have not been studied in any salmonid cell lines. To study the expression dynamics of SAA protein in the RTS-11 cell line, we first stimulated the cells with IL-1β or PBS (control) at 4, 6, 8, 10, 12, 24, 36, 48, and 72 h post-stimulation. Cell supernatants were collected, and extracellular SAA was first analysed using immunoblot. Equal volumes of supernatants from IL-1β stimulation and control were analysed along with rainbow trout rSAA as a positive control. There were high levels of endogenous rainbow trout SAA detected in the expected ~12 kDa size range in IL-1β-stimulated RTS-11 cell supernatants at between 24 and 72 h post-stimulation, with no detectable SAA in control cell supernatants (Figure 6A,B). Our reported high SAA protein expression following IL-1β stimulation is in line with previous reports on the transcriptomic level [34,35].

In order to study the expression levels of SAA at earlier timepoints, we developed a sensitive indirect ELISA. As expected, the control cells showed very low optical density (OD) signal, which remained constant between 4 and 24 h-post stimulation (Figure 6C). IL-1β-stimulated RTS-11 cells showed a trend of increased SAA signal as early as 4 h post-stimulation. The OD of the SAA kept increasing in IL-1β-stimulated cells with increased time post-stimulation, presenting an over 20-fold increase compared to control cells after 24 h (Figure 6C). To quantify the SAA protein levels, we generated a calibration curve using known concentrations of the rainbow trout rSAA protein and fitted the data into one phase exponential association (Figure 6D). The control cells showed low (mean 0.41 ng; range: 0.40–0.42 ng) protein expression suggesting SAA is continuously expressed in low levels in RTS-11 cell (Figure 6E). SAA levels increased from 0.42 ng (range: 0.41–0.43 ng) 4 h post-stimulation to 0.50 ng (range: 0.48–0.53 ng) 6 h post-stimulation, reaching 8.8 ng (range: 7.78–9.43 ng) 24 h post-stimulation with IL-1β (Figure 6E). Our data show, for the first time, (1) endogenous salmonid SAA monomer detected in the expected ~12 kDa size range; and (2) detectable SAA protein synthesis as early as 6 h post-stimulation continuously increasing before peaking at 36–48 h after IL-1β stimulation following SAA degradation. SAA levels remained very low and close to the limit of detection in control RTS-11 cell supernatants throughout the 4–72 h stimulation.

### 3.7. Localisation and Analysis of SAA Using IHC in Rainbow Trout Liver following Bacterial Pathogen Challenge with Aeromonas Salmonicida

**Figure 7:**
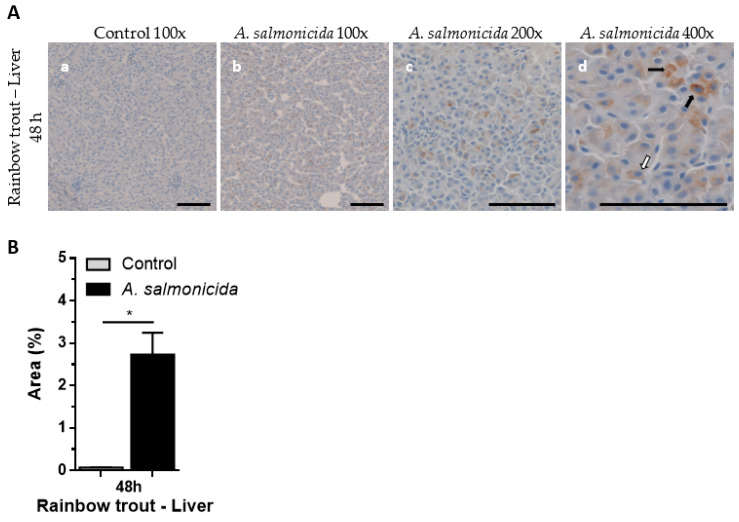
Localisation and analysis of SAA in rainbow trout liver using IHC. Fish were challenged with *A. salmonicida* or PBS (control). Liver samples were collected 48 h post-challenge, and IHC was performed using anti-SAA A10 antibody. (**A**) Representative images of control (100× magnification (**a**)) and *A. salmonicida*-challenged fish (100× (**b**), 200× (**c**) and 400× magnification (**d**)) are shown. Scale bars represent 100 μm. Black arrows represent granulated cytoplasmic SAA, while white arrows denote homogenous SAA staining. (**B**) Area (%) covered by SAA staining measured at 200× magnification. Mann–Whitney test, mean with SD, *n* = 4. * *p* < 0.05.

IHC analysis of the expression of rainbow trout SAA was performed to visualise the SAA-expressing cells in liver cross sections 48 h post-*A. salmonicida* or PBS (control) challenge. Moderate and consistent SAA signals were observed in *A. salmonicida*-challenged fish, while there was no signal in control rainbow trout tissue samples (Figure 7A), suggesting the specificity and no cross-reactivity of the SAA A10 antibody. Liver tissue samples from *A. salmonicida*-challenged fish showed two types of intracellular SAA staining: granulated and homogenous. Infected fish showed 2.73% (SD = 0.51) area covered by SAA-positive stain, while control fish had significantly lower coverage background signal, 0.07% (SD = 0.01) (Figure 7B). The data suggest that the anti-SAA antibody can distinguish between *A. salmonicida*-challenged and uninfected rainbow trout liver samples 48 h post-infection based on SAA protein synthesis.

## 4. Discussion

Our study is the first to develop and characterise a monoclonal antibody against Atlantic salmon SAA-5. The antibody A10 was used to evaluate the protein expression of SAA both in vitro and in vivo in Atlantic salmon and rainbow trout. Stimulation of flagellin, a potent pathogen-associated molecular pattern (PAMP), in Atlantic salmon resulted in very high SAA expression in liver and moderate expression in spleen in line with the transcript levels observed in rainbow trout after flagellin stimulation [18].

Furthermore, SAA levels were highly elevated in the stimulated rainbow trout monocyte/macrophage-like spleen RTS-11 cell line, and moderately elevated in the *A. salmonicida*-stimulated rainbow trout liver samples following bacterial infection. The SAA A10 antibody performed very well in a range of techniques, including ELISA, immunoblotting, and IHC, suggesting versatility in the newly developed antibody to study SAA in salmonids.

In silico analysis of the SAA peptide immunogen showed sequence similarity exclusively within SAA proteins, suggesting target uniqueness and no cross-reactivity to other proteins in salmon and trout. Surprisingly, there were multiple amino acid differences among salmonids at the SAA C-terminus (Table 1, Figure S1), the region used for the antibody development. There are three SAA proteins (SAA1, SAA2, and SAA4) reported in humans [8,36] and four in mice (SAA1, SAA2, SAA3, and SAA4) [37,38,39]. However, there are only three different SAA-annotated genes in Atlantic salmon [32,33], and four annotated SAA genes in rainbow trout [3,40,41]. Considering the gene complexity and whole-genome duplication in salmonids [42], it is reasonable to speculate that there will be new SAA proteins discovered. It is therefore plausible to conclude that the newly developed antibody detects multiple SAAs.

The linear antibody binding epitope size is short, ranging from 4 to 12 amino acids [43]. Considering the sequence differences at the 11-amino-acid-long SAA C-terminus, the antibody’s reactivity was tested with a developed recombinant Atlantic salmon SAA and a recombinant rainbow trout SAA protein. Immunoblots and mass spectrometry data (Table S1) confirmed that the SAA A10 antibody recognises recombinant Atlantic salmon SAA-5 (Figure 2), recombinant rainbow trout SAA, and endogenous rainbow trout SAA (Figure 5, Table S1).

IHC analysis showed that SAA protein was highly expressed in flagellin-stimulated Atlantic salmon liver (Figure 3) and moderately overexpressed in the spleen (Figure 4). Higher SAA levels are expected in liver than in spleen, as SAA is predominantly synthesised in hepatocytes as an acute-phase reactant protein. *A. salmonicida*-challenged rainbow trout liver showed moderate levels of SAA, whilst no SAA was detected in control fish, suggesting specificity and no cross-reactivity with other proteins in IHC (Figure 7). Increased SAA expression in liver was in line with increased SAA overexpression in the *A. salmonicida*-challenged fish [19] and SAA overexpression in flagellin-challenged rainbow trout reported before [14]. In both Atlantic salmon stimulation and rainbow trout challenge, we observed IHC two types of SAA-expressing patterns: granulated and homogenous stained SAA cells, with the granulated form becoming dominant over time. Such an observation has been previously noted in rainbow trout liver cells [14], suggesting that SAA is expressed in specific cytoplasmic compartments. The notable differences in SAA synthesis in Atlantic salmon and river trout liver cells stems from multiple factors, including differences in stimulation, its concentration, differences in immune responses between Atlantic salmon and rainbow trout, and differences among individual fish in each group.

The rainbow trout monocyte/macrophage-like RTS-11 cell line has been widely used to study immune gene expression [44,45], including *SAA* transcripts [34,35]. Our study is the first to characterise secreted SAA protein in a salmonid cell line. The levels of SAA were significantly elevated as early as 6 h post-IL-1β stimulation (Figure 6), suggesting rapid SAA synthesis and the potency of the stimulant. The SAA signal peaked at 36–48 h following a decrease 72 h post-stimulation, indicating SAA degradation. This work opens new avenues for future research with focus on SAA synthesis using a range of immunostimulants and comparing the resulting transcriptome and proteome. Furthermore, SAA is known to modulate the immune response in humans and mice [46]; therefore, it may have the same effect within salmonids. The newly developed recombinant rainbow trout and Atlantic salmon SAA proteins could serve as tools for studies depicting SAA-induced immunoregulation in salmonids.

This study did not show detectable circulating SAA in rainbow trout and Atlantic salmon sera samples. This is not surprising, as previous studies suggest low circulating SAA levels in plasma [10,16]. Additionally, SAA association with HDL makes its detection in blood a challenging task [10,14]. Low levels of SAA and its association with HDL can at least partly explain why the SAA was not detected in unprocessed sera samples in this study. Nonetheless, the antibody might be useful in detecting dissociated SAA from HDL complexes. If future studies show differences in circulating free SAA levels in healthy and infected fish, then the antibody has a potential to be developed into a non-invasive assay monitoring fish health using blood samples.

During this study, we developed a specific and versatile anti-SAA antibody that was implemented in analysing SAA protein levels in salmonids using an array of methods, including ELISAs, immunoblots, and IHC. SAA protein levels were significantly elevated in stimulated and challenged samples compared with unstimulated samples, which include Atlantic salmon liver and spleen tissues, RTS-11 cell line supernatants, and rainbow trout liver tissue. This SAA A10 antibody is a convenient and promising tool that can be used for acute-phase response research and for potential diagnostic assay development to monitor health in wild and farmed fish.

## Figures and Tables

**Table 1:** Atlantic salmon SAA-5 (Uniprot B9EPA2) amino acid sequence alignment of the peptide immunogen (RYRPNGLPRNY) in salmonids. The full SAA amino acid sequence alignment in salmonids is shown in Figure S1.

Query	RYRPNGLPRNY	
ACM09349.1	...........	serum amyloid A-5 protein [*Salmo salar*]
NP_001140037.1	...........	serum amyloid A-5 protein precursor [*Salmo salar*]
XP_029577060.1	...........	serum amyloid A-5 protein-like [*Salmo trutta*]
XP_029577059.1	........W..	serum amyloid A-5 protein-like [*Salmo trutta*]
XP_029623550.1	.........K.	serum amyloid A-5 protein-like [*Salmo trutta*]
CAA67766.1	.F..Q...K..	acute-phase serum amyloid A (SAA) [*Oncorhynchus mykiss*]
NP_001117908.1	.F..Q...K..	serum amyloid A protein precursor [*Oncorhynchus mykiss*]
CDR00160.1	.F..Q...K..	unnamed protein product [*Oncorhynchus mykiss*]
XP_021442123.1	.F..Q...KK.	serum amyloid A-5 protein [*Oncorhynchus mykiss*]
XP_035643137.1	.F..Q...K..	serum amyloid A-5 protein-like [*Oncorhynchus keta*]
XP_035595418.1	.F..Q...KK.	serum amyloid A-5 protein-like [*Oncorhynchus keta*]
XP_020335201.1	.F..Q...K..	serum amyloid A-5 protein-like [*Oncorhynchus kisutch*]
XP_020335202.1	.F..Q...K..	serum amyloid A-5 protein-like [*Oncorhynchus kisutch*]
XP_020334223.1	.F..Q...K..	serum amyloid A-5 protein [*Oncorhynchus kisutch*]
XP_046201745.1	.F..Q...N..	serum amyloid A-5 protein-like [*Oncorhynchus gorbuscha*]
XP_046205133.1	.F..Q...KK.	serum amyloid A-5 protein-like [*Oncorhynchus gorbuscha*]
XP_024274892.1	.F..Q...K..	serum amyloid A-5 protein [*Oncorhynchus tshawytscha*]
XP_029495343.1	.F..Q...K..	serum amyloid A-5 protein-like [*Oncorhynchus nerka*]
XP_029495683.1	.F..Q...K..	serum amyloid A-5 protein-like [*Oncorhynchus nerka*]
XP_041697366.1	....K...K..	serum amyloid A-5 protein [*Coregonus clupeaformis*]
XP_041715780.1	....K...KK.	serum amyloid A-5 protein [*Coregonus clupeaformis*]
CEG62717.1	....K...KK.	serum amyloid A [*Coregonus maraena*]
CAB1339290.1	H...K...KEH	unnamed protein product [*Coregonus* sp. ‘*balchen*’]
XP_041726121.1	H...K...KKH	serum amyloid A [*Coregonus clupeaformis*]
XP_038851242.1	.........K.	serum amyloid A-5 protein-like [*Salvelinus namaycush*]
XP_038851234.1	.........K.	serum amyloid A-5 protein-like [*Salvelinus namaycush*]
XP_038838543.1	.........K.	serum amyloid A-5 protein-like [*Salvelinus namaycush*]
XP_023851415.1	.F..Q...KK.	serum amyloid A-5 protein-like [*Salvelinus alpinus*]
XP_023995671.1	.........K.	serum amyloid A-5 protein-like [*Salvelinus alpinus*]

## Data Availability

The data presented in this study are contained within the article and supplementary material.

## Supplementary Materials

The following supporting information can be downloaded at: https://www.mdpi.com/article/10.3390/cells12162097/s1: Figure S1: SAA-5 amino acid sequence alignment in salmonids; Table S1: RTS-11 proteomic analysis.
