# Supplementary material for: Catch of the Day: New Serum Amyloid A (SAA) Antibody Is a Valuable Tool to Study Fish Health in Salmonids
Source: Cells. 2023 Aug 19;12(16):2097. doi: 10.3390/cells12162097 (PMC10453338; doi:10.3390/cells12162097)
Supplement: Supplementary file 1 [file cells-12-02097-s001.zip › Figure S1.pdf]

Query: LOCUS: BT057477 652 bp mRNA
Program: BLASTX
Database: nr All non-redundant GenBank CDS translations+PDB+SwissProt+PIR+PRF excluding environmental samples from WGS projects

Query range 1: 1 to 128

```
Query           60   KFVASASMKlllaglvltlvvgaqaqWYRFPGEAARGAKDMWRAYGDMKDANWKNSDKYFHARGNYDaarrgpggrwaaaVISNGREMVQGSSGRGHEDSAADQEANRWGRNGGDPNRYRPNGLPRNY  443
NP_001140037.1  20                             ......................................................................................................  121 serum amyloid A-5 protein precursor [Salmo salar]
XP_029577060.1  20                             .............................................................R........................................  121 serum amyloid A-5 protein-like [Salmo trutta]
XP_029577059.1  20                             ........V....................................................R.....................................W..  121 serum amyloid A-5 protein-like [Salmo trutta]
XP_029623550.1  20                             ..H.....VQ..R......D........H........................T..............................................K.  121 serum amyloid A-5 protein-like [Salmo trutta]
NP_001117908.1  13                             .........Q...........................................T........I...N...........K..H..........F..Q...K..  114 acute phase serum amyloid A (SAA) [Oncorhynchus mykiss]
NP_001117908.1  20                             .........Q...........................................T........I...N...........K..H..........F..Q...K..  121 serum amyloid A protein precursor [Oncorhynchus mykiss]
CDR00160.1      15   R..................................Q...........................................T........I...N...........K..H..........F..Q...K..  142 unnamed protein product [Oncorhynchus mykiss]
XP_021442123.1  20                             ............R........................................T...D..................................F..Q...KK.  121 serum amyloid A-5 protein [Oncorhynchus mykiss]
XP_035643137.1  20                             .....................................................T......................................F..Q...K..  121 serum amyloid A-5 protein-like [Oncorhynchus keta]
XP_035595418.1  20                             ..H..................................................T...D..................................F..Q...KK.  121 serum amyloid A-5 protein-like [Oncorhynchus keta]
XP_020335201.1  20                             ............................................................................................F..Q...K..  121 serum amyloid A-5 protein-like [Oncorhynchus kisutch]
XP_020335202.1  20                             ............R...............................................................................F..Q...K..  121 serum amyloid A-5 protein-like [Oncorhynchus kisutch]
XP_020334223.1  20                             ..H.........R........................................T...D..................................F..Q...K..  121 serum amyloid A-5 protein [Oncorhynchus kisutch]
XP_046201745.1  20                             .....................................................T......................................F..Q...N..  121 serum amyloid A-5 protein-like [Oncorhynchus gorbuscha]
XP_046205133.1  20                             ..H.........R........................................T...D..................................F..Q...KK.  121 serum amyloid A-5 protein-like [Oncorhynchus gorbuscha]
XP_024274892.1  20                             ............G........................................T........................K.............F..Q...K..  121 serum amyloid A-5 protein [Oncorhynchus tshawytscha]
XP_029495343.1  20                             .....................................................T........................Q........R....F..Q...K..  121 serum amyloid A-5 protein-like [Oncorhynchus nerka]
XP_029495683.1  20                             .........Q..R........................................T...D..................................F..Q...K..  121 serum amyloid A-5 protein-like [Oncorhynchus nerka]
XP_041697366.1  20                             .....................................................G...DA...................A................K...K..  121 serum amyloid A-5 protein [Coregonus clupeaformis]
XP_041715780.1  20                             ........................................N............G...DA...................A................K...KK.  121 serum amyloid A-5 protein [Coregonus clupeaformis]
CEG62717.1      20                             .....................................................G...DA...................A................K...KK.  121 serum amyloid A [Coregonus maraena]
CAB1339290.1    2      .T..G..V.........................H..I......D..R.S...H........................E...DA..RF...............V..H.........H...K...KEH  127 unnamed protein product [Coregonus sp. 'balchen']
XP_041726121.1  20                             .........H..I......D..R.S...H........................E...DA..RF...............V..H.........H...K...KKH  121 serum amyloid A [Coregonus clupeaformis]
XP_038851242.1  20                             ..H................D.................................T...D..........................................K.  121 serum amyloid A-5 protein-like [Salvelinus namaycush]
XP_038851234.1  20                             ..H.....VQ.........D.................................T...D..........................................K.  121 serum amyloid A-5 protein-like [Salvelinus namaycush]
XP_038838543.1  20                             ..H.....VQ.........D.................................T...D...R......................................K.  121 serum amyloid A-5 protein-like [Salvelinus namaycush]
XP_023851415.1  20                             ............T........................................T...D....................Q.............F..Q...KK.  121 serum amyloid A-5 protein-like [Salvelinus alpinus]
XP_023995671.1  20                             ..H................D.................................T...X...X................Z.....................K.  121 serum amyloid A-5 protein-like [Salvelinus alpinus]
```

MKlllaglvltlvvgaqaq - signal peptide
RYRPNGLPRNY - immunogen peptide

**Figure S1:** SAA5 amino acid sequence alignment in salmonids.
